# Incidence and risk factors for recurrent cardiovascular disease in middle-eastern adults: a retrospective study

**DOI:** 10.1186/s12872-019-1231-z

**Published:** 2019-11-11

**Authors:** Romona D. Govender, Saif Al-Shamsi, Elpidoforos S. Soteriades, Dybesh Regmi

**Affiliations:** 1 College of Medicine and Health Sciences, Department of Family Medicine, United Arab Emirates University, P.O. Box 17666, Al Ain, United Arab Emirates; 2 College of Medicine and Health Sciences, Department of Internal Medicine, United Arab Emirates University, Al Ain, United Arab Emirates; 3 College of Medicine and Health Sciences, Institute of Public Health, United Arab Emirates University, Al Ain, United Arab Emirates

**Keywords:** Incidence rate, Recurrent CVD events, Cardiovascular risk factors, Sex, United Arab Emirates

## Abstract

**Background:**

Individuals with established cardiovascular disease (CVD) and risk factors such as age, smoking, hypertension, and diabetes mellitus are at an increased risk of recurrent cardiovascular events and death. The incidence rate of recurrent CVD events varies between countries and populations. The United Arab Emirates (UAE) has one of the highest age-standardized death rates for CVD worldwide. The aim of our study was to estimate the incidence rates and determine the predictors of recurrent CVD events among UAE nationals.

**Methods:**

We investigated an outpatient-based cohort of patients with a history of CVD visiting Tawam Hospital between April 1, 2008 and December 31, 2008. They were followed-up until July 31, 2018. Univariable and multivariable Cox proportional hazards regression models were used to determine the association between major CVD risk factors and the risk of CVD recurrence.

**Results:**

A total of 216 patients (167 males, 49 females) with a history of CVD were included. They were followed for a median (interquartile range) of 8.1 (5.5–9.3) years, with a total of 1184 patient-years of follow-up. The overall incidence rate of recurrent CVD events was 92.1 per 1000 patient-years. The 8-year cumulative incidence was 73.7%. Age, female sex, and diabetes mellitus were significant predictors of recurrent CVD events, where females had a 1.96 times higher risk of recurrent CVD events than males.

**Conclusion:**

Significant predictors of recurrent CVD events are older age, female sex, and diabetes mellitus. The incidence rate of recurrent CVD events was 92.1 per 1000 patient-years. Preventive measures, based on international guidelines for CVD management, may improve CVD morbidity and mortality in the UAE population.

## Background

Individuals with a history of cardiovascular disease (CVD) are at a much higher risk of recurrent CVD events or death [1]. Nonetheless, the prognosis of CVD has improved due to developments in early revascularization, antithrombotic medications, and other tertiary prevention measures, including the control of major CVD risk factors [2]. The incidence rate of recurrent CVD events varies between countries and populations. The Second Manifestations of ARTerial disease study conducted by the University Medical Centre Utrecht demonstrated a 53% reduction in the crude incidence rate of recurrent CVD events between 1996 and 2014 [3]. Studies from the United States and Western Australia have also documented decreasing trends in incidence and mortality rates [4–6]. However, the increasing prevalence of CVD risk factors e.g. obesity and diabetes mellitus [2], and the emergence of novel biochemical risk factors, such as cellular, imaging parameters [7] and C-reactive protein [8] are strong predictors of cardiovascular events, all of which are now challenging these declining trends.

Independent predictors of recurrent CVD events or death include age, smoking, hypertension (HTN), dyslipidemia, diabetes mellitus, chronic kidney disease, and the underutilization of medications recommended by current treatment guidelines [9, 10]. A previous study conducted in the United Arab Emirates (UAE) reported 35% with HTN, 34% having dyslipidemia, 14.4% had a history of coronary artery disease (CAD) and 29.5% with diabetes mellitus also had concomitant macrovascular complications [11]. The UAE has one of the highest age-standardized death rates for CVD, at 204 and 309 per 100,000 [12], when compared to North America, which has a rate of 143 and 204 per 100,000, and Western Europe, which has a rate of 132 and 187 per 100,000 in females and males, respectively [13].

The UAE is experiencing an economic transition since the discovery of oil over 5 decades ago, and as a consequence, there has been a change in lifestyle among UAE populations that has resulted in an increase in CVD risk factors and worsening outcomes. To the best of our knowledge, few studies have investigated the epidemiology of recurrent CVD events in the UAE. Thus, we aimed to assess the incidence rate of recurrent CVD events in males and females and evaluated the influence of major CVD risk factors on the recurrence rate of CVD events among UAE nationals.

## Methods

### Study setting

This retrospective cohort study was conducted in Tawam Hospital in Al Ain City, which is the fourth largest city in the UAE, with a population of 650,000. Approximately 30% of this population are UAE nationals. Tawam Hospital is one of the largest tertiary care hospitals in Al Ain, providing health care services to UAE nationals and the expatriate community.

The United Arab Emirates University and Tawam Hospital Review Boards (CRD 239/13) granted ethical approval for this study. The Cerner© electronic medical record (EMR) management system was utilized to obtain patient data retrospectively. Patient information was anonymized to protect patients’ identities. Informed consent was waived as identifying data was not collected from the EMR.

### Study population

The original study population included all patients who visited the outpatient clinics of Tawam Hospital between April 1, 2008 and December 31, 2008. Patients who were ≥ 18 years old and had an established diagnosis of coronary artery disease (CAD), cerebrovascular disease, or peripheral vascular disease (PVD) were included in this study. Established CAD was defined as a documented history of stable angina, unstable angina, percutaneous coronary intervention (PCI), coronary artery bypass graft surgery, or myocardial infarction (MI). Cerebrovascular disease was defined as a documented diagnosis of transient ischemic attack (TIA) or stroke. PVD was defined as a documented diagnosis of peripheral artery disease. Patients with missing baseline data were excluded. Patients were followed-up from the time of diagnosis until a recurrent CVD event, death, or the end of the study period on July 31, 2018.

### Baseline characteristics

Data retrieved from the EMR included sociodemographic parameters such as age; sex; body mass index (BMI); CVD risk factors, included smoking, a history of HTN, systolic blood pressure (SBP), diastolic blood pressure (DBP), history of diabetes mellitus, total cholesterol, and low-density lipoprotein (LDL); and medications, such as the use of anti-hypertensive medications, lipid-lowering medications, and antiplatelet therapy. Smokers were defined as current smokers or having a history of smoking based on the results of a recent local study [14]. BMI was calculated as weight in kilograms divided by the height in meters squared. Commonly accepted BMI ranges are those recommended by the World Health Organization: overweight (BMI 25–29.99 kg/m^2^), obese class I (BMI 30–34.99 kg/m^2^), obese class II (BMI 36–40 kg/m^2^), and obese class III (≥ 41 kg/m^2^) [15]. Diabetes mellitus was defined as receiving oral hypoglycemic medications or having a diagnosis made by an attending physician. HTN was defined as receiving anti-hypertensive medications or having a SBP ≥140 mmHg or DBP ≥ 90 mmHg, and blood pressure was categorized as follows: normal (SBP < 140 or DBP < 90), grade 1 HTN (SBP 140–159 or DBP 90–99), grade 2 (SBP 160–179 or DBP 100–109), and grade 3 (SBP ≥180 or DBP ≥110) [16]. Based on Adult Treatment Panel III guidelines, a LDL level < 2.6 mmol/L (< 100 mg/dL) was classified as optimal and ≥ 2.6 mmol/L (≥100 mg/dL) as suboptimal, and total cholesterol ≤5.17 mmol/L (≤200 mg/dL) was classified as optimal and > 5.17 mmol/L (> 200 mg/dL) as suboptimal [17].

### Recurrent CVD events

The outcome of our study included recurrent CVD events and/or death. Recurrent CVD events included stable angina, unstable angina, PCI, MI, TIA, and cerebral infarction (confirmed by computed tomography scan or magnetic resonance imaging), or ankle-brachial index < 0.9. Vascular death was defined as sudden death or death from a fatal MI or fatal stroke. The data on patients’ comorbidities and CVD events were collected using the histories recorded on the EMR.

The overall incidence rate of recurrent CVD events, the incidence rates in males and females, and the incidence rates according to different age categories were calculated by dividing the number of study participants with recurrent CVD events by the total patient-years of follow-up for the study population and for each subgroup accordingly.

### Statistical analysis

The study’s sample size was calculated using a formula for a study designed to estimate incidence in a population [18]. A sample size of 194 was determined based on an anticipated 60% incidence of recurrent CVD events [19] and utilizing 80% power with a 2-sided significance level of 0.05.

Baseline variables were compared between males and females using independent-samples t-test for continuous variables and Fisher’s exact test (2-tailed) for categorical variables. Continuous variables are presented as means [±standard deviation (SD)], while categorical variables are presented as proportions. The patient-years at risk for recurrent CVD events were calculated for each subject from the baseline visit until the occurrence of a recurrent CVD event, death, or the last outpatient clinic visit, whichever occurred first. Univariable and multivariable Cox proportional hazards regression models evaluated the risk factors for recurrent CVD events. The independent predictors included in the multivariable analysis were age (continuous), sex, smoking history, blood pressure (systolic and diastolic), anti-platelet medication, diabetes mellitus, LDL (continuous), and BMI (continuous). Proportional hazards assumption was assessed by log-log plots, while multi-collinearity was evaluated by examining the tolerance value. The results are expressed as hazard ratios and 95% confidence intervals (CIs). A *p*-value < 0.05 (2-sided) was considered statistically significant for all tests. All statistical analyses were performed using IBM SPSS software, version 25 (IBM Corporation, Armonk, NY, USA).

## Results

A total of 216 patients (167 males, 49 females) with a history of CVD at baseline were included in our study. They were followed-up for a median (interquartile range) of 8.1 (5.5–9.3) years, with a total of 1184 patient-years of follow-up. Mean age was 65.7 (SD: 11.2, range: 28–96) years. At baseline, CAD, cerebrovascular disease, and PVD were diagnosed in 69, 38, and 6.5% of the patients, respectively. Over the 8-year follow-up period, at least 51% of patients (79 males, 30 females) had experienced a recurrent CVD event, with 9 (4.2%), 66 (30.6%), and 26 (12.0%) patients having developed PVD, CAD, and cerebrovascular disease, respectively.

The overall incidence rate of recurrent CVD events was 92.1 per 1000 patient-years of follow-up (95% CI: 76.0–110.6). Assuming a constant incidence rate over time, the 8-year cumulative incidence of recurrent CVD events was 73.7%. The annual CVD mortality rate over the study period was 18.5 per 1000 individuals (95% CI: 13.4–25.2) per year. The incidence rates of recurrent CVD events among females and males were 119.8 (95% CI: 82.3–168.9) and 84.6 (95% CI: 67.4–104.9) per 1000 patient-years of follow-up, respectively. The incidence rates for different age categories were as follows: 51.1, 35.5, 68.4, 70.8, and 115.2 for patients aged ≤34, 35–44, 45–54, 55–64, and ≥ 65 years per 1000 years of follow-up, respectively.

Table 1 shows the sociodemographic and risk factor characteristics of our study population. At baseline, 3.7% of patients aged 35–44 years had CVD, and this was even higher, at 7.4%, in patients aged 45–54 years. A sizeable portion of the population were smokers (27%) despite having a history of CVD. Furthermore, 94.9% of our patients were hypertensive, and about 43% of them had elevated BP values with 31.5, 8.3, and 3.2% of patients diagnosed with HTN grade 1, 2, and 3, respectively. Of the 85% on statins, at least half of our patients had elevated LDL levels. An extremely high percentage of patients with diabetes mellitus (62.5%) was noted in our cohort. Furthermore, two-thirds of the study population was either overweight (38.4%) or obese (27.3%). Notably, the prevalence of obesity among female patients with CVD was 41%.

**Table 1:** Baseline characteristics of the study population (*N* = 216)

Sociodemographics and other risk factors	Total *n* (%)	Sex	
		Female 49 (23%)	Male 167 (77%)
Age (mean ± SD)	65.7 ± 11.2	65.0 ± 10.6	65.9 ± 11.4
Age, years			
< 34	4 (1.9)	0	4 (2.4)
35–44	8 (3.7)	2 (4.1)	6 (3.6)
45–54	16 (7.4)	7 (14.3)	9 (5.4)
55–64	62 (28.7)	14 (28.6)	48 (28.7)
≥ 65	126 (58.3)	26 (53.1)	100 (59.9)
Smoking			
Yes	58 (26.9)	1 (2.0)	57 (34.1)
Hypertension			
Yes	205 (94.9)	47 (95.9)	158 (94.6)
Hypertension grade			
Normal	123 (56.9)	26 (53.1)	97 (58.1)
Grade 1	68 (31.5)	16 (32.7)	52 (31.1)
Grade 2	18 (8.3)	3 (6.1)	15 (9.0)
Grade 3	7 (3.2)	4 (8.2)	3 (1.8)
Diabetes mellitus			
Yes	135 (62.5)	28 (57.1)	107 (64.1)
Total cholesterol, mmol/L			
> 5.17	44 (20.4)	13 (26.5)	31 (18.6)
LDL cholesterol, mmol/L			
≥ 2.6	109 (50.5)	28 (57.1)	81 (48.5)
BMI, mean ± SD	27.0 ± 5.1	29.1 ± 5.3	26.4 ± 4.9
BMI, kg/m^2^			
≤ 24.99	74 (34.3)	10 (20.4)	64 (38.3)
25–29.99	83 (38.4)	19 (38.8)	64 (38.3)
30–34.99	43 (19.9)	11 (22.4)	32 (19.2)
> 35	16 (7.4)	9 (18.4)	7 (4.2)
Anti-platelet medication			
Yes	113 (52.3)	27 (55.1)	86 (51.5)
Statins			
Yes	184 (85.2)	40 (81.6)	144 (86.2)
Persons-years of follow-up	1184.0	250.4	933.6
Recurrent CVD events	109	30	79

*CVD* cardiovascular disease, *LDL* low-density lipoproteins, *BMI* body mass index, *SD* standard deviation

Table 2 shows the univariable and multivariable analyses determining the association between different major CVD risk factors and the risk of recurrent CVD events. The proportional hazards assumption was not significant, and the tolerance ranged from 0.58–0.97, which indicated an absence of multicollinearity. Older age, female sex, and diabetes mellitus were significant predictors of recurrent CVD events or death in the multivariable model, and females had a 1.96 times higher risk for recurrent CVD events than males. Sex and diabetes mellitus, although not significant in the univariable analysis, became significant in the multivariable analysis. We, therefore, tested for an interaction effect in the multivariable model. However, no significant interaction between sex and diabetes mellitus was observed (*p* = 0.547).

**Table 2:** Hazard ratios of risk factors for recurrent CVD events (N=216)

Risk factor	HR (95% CI)					
	Univariable model	*p* value	Multivariable model*	*p* value	Multivariable model**	*p* value
Age (continuous)	1.03 (1.01–1.05)	0.001	1.04 (1.02–1.06)	0.001	1.04 (1.02–1.06)	< 0.001
Age (categorical), years						
< 34	Ref					
35–44	0.66 (0.06–7.28)	0.733				
45–54	1.29 (0.15–10.73)	0.815				
55–64	1.34 (0.18–9.88)	0.774				
≥ 65	2.24 (0.31–16.03)	0.428				
Sex						
Male	Ref		Ref		Ref	
Female	1.40 (0.92–2.14)	0.115	1.96 (1.22–3.15)	0.005	1.55 (1.01–2.37)	0.043
Smoking						
No	Ref		Ref			
Yes	1.01 (0.67–1.53)	0.958	1.26 (0.80–1.99)	0.319		
SBP	1.01 (1.00–1.02)	0.156	1.01 (1.00–1.02)	0.166		
DBP	1.00 (0.98–1.01)	0.689	0.99 (0.97–1.02)	0.581		
Hypertension grade						
Normal	Ref					
Grade 1	0.86 (0.56–1.33)	0.506				
Grade 2	2.47 (1.37–4.43)	0.003				
Grade 3	1.49 (0.54–4.12)	0.439				
Diabetes mellitus						
No	Ref		Ref		Ref	
Yes	1.45 (0.97–2.18)	0.072	2.04 (1.29–3.22)	0.002	1.79 (1.17–2.73)	0.007
LDL (continuous)	0.96 (0.80–1.15)	0.634	0.99 (0.82–1.19)	0.927		
LDL (categorical), mmol/L						
< 2.6	Ref					
≥ 2.6	0.94 (0.65–1.38)	0.767				
BMI (continuous)	0.98 (0.94–1.02)	0.228	0.96 (0.92–1.00)	0.065		
BMI (categorical), kg/m^2^						
≤ 24.9	Ref					
25–29.9	0.98 (0.63–1.506)	0.910				
30–34.9	0.55 (0.31–0.98)	0.044				
35+	1.43 (0.73–2.81)	0.293				
Anti-platelet medication						
No	Ref		Ref			
Yes	0.85 (0.58–1.23)	0.380	0.73 (0.50–1.08)	0.114		

*Adjusted for age (continuous), sex (dichotomous), smoking (dichotomous), SBP, DBP, anti-platelet medication, diabetes mellitus (dichotomous), LDL cholesterol (continuous), and BMI (continuous)

**Adjusted for age (continuous), sex (dichotomous), diabetes mellitus (dichotomous)

*BMI* body mass index, *LDL* Low-density lipoprotein, *SBP* systolic blood pressure, *DBP* diastolic blood pressure (continuous)

**Fig. 1:**
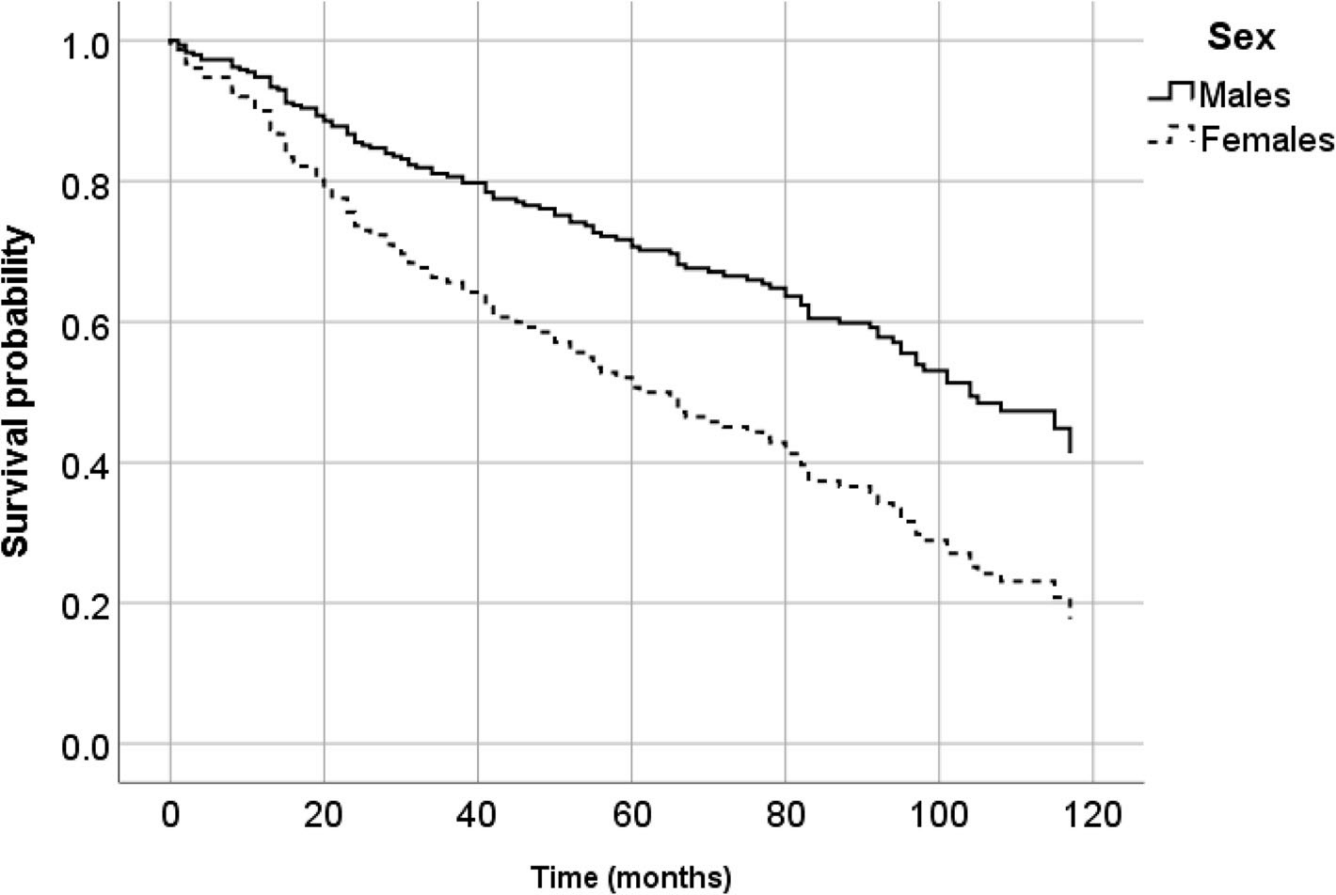
Adjusted Cox regression survival curve for recurrent CVD events by sex

A multivariable survival curve comparing the incidence of CVD events in males and females is shown in Fig. 1. We found that for every 1-year increase in age, patients had a 4% higher probability of developing a recurrent CVD event. Furthermore, a history of diabetes mellitus and prior CVD doubled the risk of recurrent CVD events.

Adjusted for age (continuous), sex (dichotomous), smoking (dichotomous), SBP, DBP, anti-platelet medication, diabetes mellitus (dichotomous), LDL cholesterol (continuous), and BMI (continuous).

## Discussion

To the best of our knowledge, this is the first study to examine the incidence rates and explore the risk factors for recurrent CVD events among UAE nationals. The overall incidence rate of recurrent CVD events in this study (92.1 per 1000 patient-years) appears to be one of the highest reported rates in literature [19–22] and in the Middle Eastern region [23]. Older age, female sex and diabetes mellitus were found to be significant risk predictors of recurrent CVD events or death. One of the intriguing findings in our study was the significantly higher risk of recurrent CVD events among females than among males. In addition, about 43% of the patients had poorly controlled blood pressures with 31.5, 8.3, and 3.2% of patients diagnosed with HTN grade 1, 2, and 3, respectively, and despite the majority of patients on prescribed statins, only half had optimal LDL levels.

In this study, recurrent CVD increased exponentially with advancing age, which was supported by the findings of previous studies [5, 21, 23, 24]. For every 1-year increase in age, we found a 4% higher probability for recurrent CVD events in the UAE population, whereas the probability was 2% lower in a neighboring country [23]. Moreover, similar to the findings of Giorda et al., we found that diabetes mellitus was a significant independent risk factor for recurrent CVD events [24]. This study found that 62.5% of patients had concurrent diabetes mellitus and CVD, which was almost double (29.5%) of that found in a study that was conducted in the same city as ours [11]. After adjusting for all risk factors, the probability of CVD recurrence in patients with diabetes mellitus was 2 times higher than that in patients without diabetes mellitus. These findings were similar to those of other studies [20, 25].

While the results of sex-based studies showed that females have more favorable results than males [26–28], other studies [29, 30] concurred with the results obtained in this study, showing that females had a 1.96 times higher risk of recurrent CVD events than their male counterparts, and thus, were more likely to have a worse CVD risk-factor profile [29, 30]. Many factors, such as an increased prevalence in HTN, diabetes, and obesity, may contribute to the poor CVD risk profiles of women [30]. Additionally, smoking, coupled with an increased consumption, had a greater negative effect in women than in men. Research on current smokers showed hazard ratios of 3.46 (95% CI: 3.02–3.98) in women compared to 2.23 (95% CI: 2.03–2.44) in men [30].

Based on the existing literature [31–34], we expected a positive association between the major modifiable risk factors (smoking, HTN, and suboptimal LDL levels) and cardiovascular outcomes. However, in our study the associations between smoking, HTN, elevated levels of LDL with recurrent CVD events were not significant. Evidence suggests that anti-hypertensive treatments [31] and lipid-lowering medications are commonly underutilized in patients at high-risk of recurrent CVD events [31–36]. Whether this is due to poor adherence to treatment or inadequate treatment regimens remains unclear and should be further investigated.

### Strengths

Our study is the first to report the incidence rates and risk factors associated with recurrent CVD events in the UAE population. The extended duration of the follow-up period, assessing recurrent CVD events and mortality, has enriched the epidemiological data for this region.

### Limitations

This study has some limitations. This is a retrospective study, which made researchers rely on the accurate capturing of clinical data by physicians on the EMRs. The relatively small sample size and the geographical distribution limits the generalizability of our results to other cohorts. Further studies should be performed using other population groups. Sociodemographic parameters such as education and occupation would have benefitted the analysis, but these were not available for inclusion. Moreover, prediction models to evaluate the effect of other medical comorbidities such as heart failure, atrial fibrillation, and chronic kidney disease which may have influenced CVD outcomes and prognosis, were not included in the analysis. Thus, future studies should include these parameters.

## Conclusions

Our study found high incidence rates of recurrent CVD events among patients with a history of CVD attending the outpatient clinics in a tertiary hospital in Al Ain. Furthermore, older age, female sex, and diabetes mellitus were strong independent predictors of recurrent CVD events. Although local screening programs are designed towards early diagnosis and the provision of disease management interventions [37], more needs to be done to improve primary and tertiary preventative measures in order to improve CVD morbidity and mortality among UAE nationals.

## Data Availability

The data used to support the findings of this study are available from the corresponding author upon reasonable request.

## Abbreviations

BMI: Body mass index
BP: Blood pressure
CAD: Coronary artery disease
CI: Confidence interval
CVD: Cardiovascular disease
DBP: Diastolic blood pressure
HTN: Hypertension
LDL-C: Low-density lipoprotein-cholesterol
MI: Myocardial infarction
PCI: Percutaneous coronary intervention
PVD: Peripheral vascular disease
SBP: Systolic blood pressure
SD: Standard deviation
TIA: Transient ischemic attack
UAE: United Arab Emirates
